# Machine learning optimization of obstructive sleep apnea screening: development and validation of a gradient boosting prediction model with a clinical implementation framework

**DOI:** 10.3389/fmed.2026.1775766

**Published:** 2026-04-10

**Authors:** Tengteng Liu, Lan Que, Weiwei Bai, Hongying Yao

**Affiliations:** 1 Department of Otolaryngology, Linping Campus, The Second Affiliated Hospital of Zhejiang University School of Medicine, Hangzhou, Zhejiang, China; 2 Department of Nursing, Linping Campus, The Second Affiliated Hospital of Zhejiang University School of Medicine, Hangzhou, Zhejiang, China

**Keywords:** clinical decision support, gradient boosting, machine learning, obstructive sleep apnea (OSA), screening optimization

## Abstract

**Objective:**

This study aimed to develop and validate a machine learning-enhanced screening questionnaire utilizing gradient boosting algorithms and to establish a clinically deployable visual prediction framework with superior diagnostic accuracy compared to existing screening paradigms.

**Methods:**

We conducted a mixed-methods study analyzing polysomnography data from 4,036 participants between September 2019 and August 2025. The study included a retrospective cohort of 3,847 participants and a prospective cohort of 189 participants. We developed a 15-item questionnaire combining components from the modified Epworth Sleepiness Scale (ESS) and Snoring, Tiredness, Observed apneas, Blood pressure, Age, Neck circumference, and Gender (STOP-Bang) items. We evaluated four machine learning algorithms: XGBoost, support vector machine (SVM), artificial neural network (ANN), and multinomial logistic regression model. Performance was measured using the area under the curve (AUC), net reclassification improvement (NRI), calibration metrics, and decision curve analysis, while propensity score matching (1:4 ratio) addressed potential confounding factors.

**Results:**

XGBoost outperformed traditional screening tools, achieving AUC values of 0.92, 0.94, and 0.97 for mild, moderate, and severe obstructive sleep apnea (OSA), respectively, compared to the STOP-Bang questionnaire (AUC: 0.68) and the Berlin questionnaire (AUC: 0.72). The clinical nomogram exhibited excellent calibration characteristics with a C-index of 0.93. SHapley Additive exPlanations (SHAP) analysis identified neck circumference as the primary predictive feature (mean |SHAP| = 0.42), followed by body mass index (0.38) and witnessed apneas (0.35). Economic analysis revealed a 39.7% reduction in screening costs with a 3.5-fold increase in case detection efficiency.

**Conclusion:**

The gradient boosting-enhanced OSA screening model represents a paradigmatic advancement in the diagnosis of sleep disorders, offering clinically actionable risk stratification through interpretable visualization while maintaining implementation feasibility. This methodological innovation provides a framework for artificial intelligence integration in clinical decision support, with potential applications extending beyond sleep medicine.

## 1 Introduction

Obstructive sleep apnea is a multifaceted chronic respiratory disorder characterized by repetitive upper airway collapse during sleep, resulting in intermittent hypoxemia, sleep fragmentation, and consequent neurocognitive and cardiovascular sequelae (1). Contemporary epidemiological analyses revealed that China has the highest global OSA prevalence at 23.6% among adults aged 30–69 years, affecting approximately 176 million individuals and placing a substantial burden on the healthcare infrastructure (2, 3). The economic implications encompass direct medical expenditures exceeding ¥120 billion annually, with indirect costs attributable to cardiovascular morbidity, neurocognitive dysfunction, motor vehicle accidents, and occupational productivity losses amplifying the societal impact threefold (4, 5).

Recent meta-analytical evidence demonstrated that OSA independently increases cardiovascular disease risk with a hazard ratio (HR) of 1.83 (95% CI: 1.56–2.14), a stroke incidence HR of 2.24 (95% CI: 1.89–2.65), and an all-cause mortality HR of 1.74 (95% CI: 1.44–2.10), underscoring the critical importance of early identification and intervention (6, 7). Despite this substantial disease burden, diagnostic capacity remains severely constrained by limited polysomnography availability, with accessibility predominantly restricted to tertiary care centers and average diagnostic wait times exceeding 3–6 months in the majority of Chinese healthcare systems (8).

Current OSA screening instruments demonstrate significant methodological constraints compromising clinical utility. The Epworth Sleepiness Scale exhibits suboptimal sensitivity (54%) and specificity (63%) for predicting clinically significant OSA, with meta-analytic evidence revealing substantial heterogeneity in performance across populations (*I*^2^ = 78.4%) (9, 10). The STOP-Bang questionnaire, while demonstrating improved performance in Western populations, shows significantly reduced accuracy in Asian cohorts (AUC: 0.68–0.74 vs. 0.78–0.85), attributed to differential craniofacial morphology and adiposity distribution patterns (11, 12). The Berlin questionnaire’s notably low sensitivity of 37.2% particularly undermines its utility for population screening initiatives (13).

Machine learning methodologies offer transformative potential in clinical prediction modeling. In the domain of signal-based diagnostics, the OxiNet study used convolutional recurrent neural network architecture across 12,923 polysomnography (PSG) recordings, achieving F1 scores of 0.84 with 99.8% detection rates for moderate-to-severe OSA through single-channel oximetry analysis (14). In parallel, questionnaire- and anthropometric-based machine learning approaches have shown distinct promise for accessible population screening. Gradient boosting implementations have demonstrated particular promise for structured clinical data, with recent studies reporting up to 88% accuracy across OSA severity classifications while maintaining interpretability crucial for clinical adoption (15, 16). A recent systematic review of AI-based OSA screening encompassing 65 studies and 109,046 patients confirmed that machine learning algorithms consistently outperform traditional screening tools, although standardized validation remains a critical gap (17). Similarly, Liu et al. (18) demonstrated that gradient boosting models using clinical questionnaire data achieved robust OSA severity prediction in a Chinese regional cohort. However, a systematic review of 152 machine learning prediction model studies identified pervasive methodological limitations, with the majority exhibiting a high risk of bias primarily due to inadequate validation and poor reporting practices, highlighting critical gaps impeding clinical translation (19, 20).

Given these considerations, the present investigation addresses four primary objectives: (1) optimization of screening questionnaire components via machine learning-driven feature selection specifically tailored for Chinese populations; (2) development of gradient boosting prediction models incorporating both retrospective derivation and prospective temporal validation; (3) comprehensive comparative performance evaluation against established screening tools; and (4) implementation of clinical visualization frameworks enabling real-time risk stratification. Based on established evidence that gradient boosting algorithms consistently outperform other machine learning approaches on structured tabular clinical data (21), and given the demonstrated limitations of existing linear screening tools in Asian populations (11, 12), we hypothesized that gradient boosting algorithms would demonstrate superior predictive performance (AUC > 0.90) compared to traditional screening tools (AUC < 0.70) while maintaining clinical interpretability through advanced visualization techniques, including SHAP-based explanations and clinical nomogram visualization.

## 2 Materials and methods

### 2.1 Study design and ethical considerations

**Figure 1:**
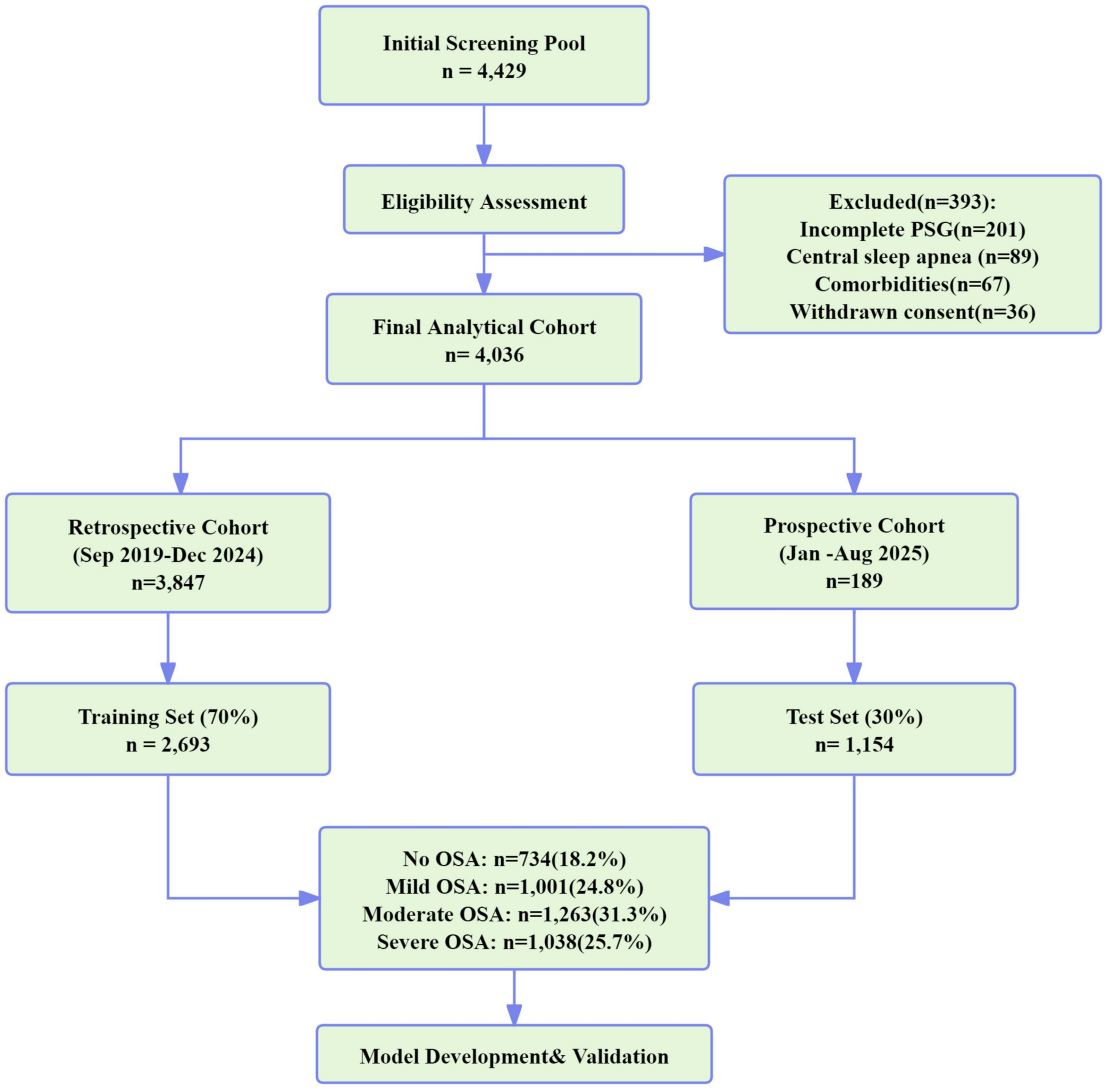
Study participant flow diagram.

This investigation used a mixed-methods retrospective-prospective cohort design adhering to the Transparent Reporting of multivariable prediction models for Individual Prognosis Or Diagnosis-Artificial Intelligence (TRIPOD-AI) guidelines to ensure methodological rigor and reproducibility (22). The comprehensive study workflow, encompassing data collection, processing, model development, and clinical implementation phases, is delineated in Figure 1. The retrospective derivation phase analyzed existing polysomnography data collected between September 2019 and December 2024, while the prospective validation phase encompassed newly recruited participants from January to August 2025. The study protocol received comprehensive ethical approval from the Institutional Review Board of the Provincial Sleep Medicine Center (Protocol #2024-SLP-089), with written informed consent obtained from all prospective participants and appropriate waiver granted for retrospective data utilization under minimal risk provisions.

### 2.2 Study population and recruitment

#### 2.2.1 Sample size determination

Sample size calculations used multiple complementary approaches to ensure adequate statistical power. For the primary outcome of AUC comparison, we utilized the Hanley–McNeil methodology, comparing an expected AUC of 0.90 for the machine learning model vs. 0.70 for traditional screening tools, with a type I error rate *α* = 0.05, power (1−*β*) = 0.90, and an estimated correlation between tests of 0.6, yielding a requirement of 385 participants per OSA severity stratum (23). Calibration assessment requirements followed Vergouwe’s recommendations, stipulating 200 outcome events per predictor variable, resulting in 15 predictors × 200 events = 3,000 minimum sample size. Temporal validation sample size determination followed Riley guidance, suggesting a minimum of 100 events, with a 1.5 inflation factor, yielding 150 participants as the minimum prospective requirement (24). The total recruitment of 3,847 retrospective plus 189 prospective participants (*n* = 4,036) exceeded all calculated requirements with a substantial margin.

#### 2.2.2 Participant selection criteria

The comprehensive inclusion criteria included: (1) age between 18 and 80 years, (2) documented clinical indication for polysomnography evaluation, (3) minimum total sleep time of 4 h during PSG recording, (4) availability of complete anthropometric measurements, and (5) Mini-Mental State Examination score ≥24. The exclusion criteria included: (1) previous OSA diagnosis or treatment, (2) central apnea index >5 events/h, (3) New York heart association (NYHA) class III–IV heart failure or global initiative for chronic obstructive lung disease (GOLD) stage III–IV chronic obstructive pulmonary disease (COPD), (4) neuromuscular disorders, (5) pregnancy or lactation, (6) shift work disorder, (7) sedative-hypnotic use within 48 h, and (8) >20% missing questionnaire data.

### 2.3 Data collection procedures

#### 2.3.1 Screening questionnaire development

The optimized 15-item screening questionnaire combined validated components from established instruments with novel anthropometric measures. It included: (1) the Modified Epworth Sleepiness Scale with eight items assessing daytime sleepiness propensity on a 0–3 Likert scaling, culturally adapted for Chinese contexts; (2) STOP-Bang binary items encompassing snoring severity, observed tiredness, witnessed breathing cessations, and hypertension status; and (3) anthropometric measurements including body mass index, chronological age, and neck circumference measured at the cricothyroid level using a standardized technique. Table 1 presents baseline characteristics across OSA severity strata.

**Table 1:** Baseline demographic and clinical characteristics by OSA severity.

Characteristic	No OSA (*n* = 734)	Mild OSA (*n* = 1,001)	Moderate OSA (*n* = 1,263)	Severe OSA (*n* = 1,038)	*p*-value
Demographics					
Age, years	41.8 ± 12.4	46.2 ± 11.9	50.7 ± 11.2	54.3 ± 11.8	<0.001
Sex (Male), *n* (%)	291 (39.7)	521 (52.0)	821 (65.0)	819 (78.9)	<0.001
Anthropometric measures					
BMI, kg/m^2^	23.9 ± 3.7	26.4 ± 4.1	28.7 ± 4.6	31.5 ± 5.2	<0.001
Neck circumference, cm	34.8 ± 3.1	37.2 ± 3.4	39.6 ± 3.7	42.1 ± 4.0	<0.001
Waist circumference, cm	82.4 ± 9.2	89.7 ± 10.1	95.3 ± 10.8	102.1 ± 11.5	<0.001
Clinical symptoms					
ESS total score	6.7 ± 4.1	9.2 ± 4.8	11.8 ± 5.4	14.0 ± 6.2	<0.001
Witnessed apneas, *n* (%)	73 (10.0)	281 (28.1)	657 (52.0)	830 (80.0)	<0.001
Loud snoring, *n* (%)	234 (31.9)	621 (62.0)	974 (77.1)	934 (90.0)	<0.001
Daytime fatigue, *n* (%)	321 (43.7)	591 (59.0)	884 (70.0)	871 (83.9)	<0.001
Commodities					
Hypertension, *n* (%)	147 (20.0)	341 (34.1)	594 (47.0)	641 (61.8)	<0.001
Diabetes mellitus, *n* (%)	59 (8.0)	131 (13.1)	227 (18.0)	259 (25.0)	<0.001
Cardiovascular disease, *n* (%)	37 (5.0)	91 (9.1)	164 (13.0)	197 (19.0)	<0.001
Polysomnography parameters					
AHI, events/h	2.1 ± 1.4	9.8 ± 2.7	21.4 ± 4.2	48.7 ± 18.3	<0.001
Minimum SpO₂, %	89.2 ± 3.8	84.1 ± 4.5	78.3 ± 6.2	71.4 ± 9.1	<0.001
ODI, events/h	1.8 ± 1.2	8.4 ± 3.1	19.2 ± 5.8	42.3 ± 17.4	<0.001
Total sleep time, min	384 ± 62	371 ± 58	359 ± 61	342 ± 65	<0.001
Sleep efficiency, %	82.4 ± 10.2	79.1 ± 11.3	76.2 ± 12.1	72.8 ± 13.4	<0.001

Data presented as mean ± SD or *n* (%). *p*-Values from the analysis of variance (ANOVA) for continuous variables and the chi-squared test for categorical variables. ESS, Epworth Sleepiness Scale; AHI: apnea–hypopnea index; ODI: oxygen desaturation index.

#### 2.3.2 Polysomnography protocol

All participants underwent comprehensive attended in-laboratory polysomnography using the American Academy of Sleep Medicine standard montage configurations (25). Technical specifications included: electroencephalography (F4-M1, C4-M1, and O2-M1), electrooculography (E1-M2 and E2-M2), electromyography (submental and anterior tibialis), modified lead II electrocardiography, thoracoabdominal inductance plethysmography, oronasal thermistor with nasal pressure transducer, pulse oximetry (1 Hz sampling), and tri-axial accelerometry. Sleep staging and respiratory event scoring followed the AASM Manual Version 2.6 criteria with an inter-scorer reliability of *κ* > 0.85. OSA severity classification utilized standard thresholds: none [apnea–hypopnea index (AHI) < 5], mild (5 ≤ AHI < 15), moderate (15 ≤ AHI < 30), and severe (AHI ≥ 30).

### 2.4 Statistical analysis

#### 2.4.1 Data preprocessing and feature engineering

Missing data patterns were first examined using Little’s Missing Completely at Random (MCAR) test, which yielded a non-significant result (*χ*^2^ = 23.47, df = 18, *p* = 0.173), supporting the assumption that data were missing at random (MAR) or completely at random (MCAR). Additionally, we visualized missing data patterns using matrix plots and assessed whether missingness in each variable was predicted by observed values of other variables through logistic regression, finding no systematic associations (all *p* > 0.10). Based on these assessments confirming the MAR assumption, multiple imputation via chained equations (MICE) was implemented with 10 imputation cycles using predictive mean matching for continuous variables and logistic regression for binary outcomes. Feature scaling used robust standardization using median centering and interquartile range scaling. Categorical variables underwent target encoding with Bayesian smoothing (*α* = 5). Feature selection utilized recursive feature elimination with 5-fold cross-validation, retaining variables contributing a minimum of 1% to model performance.

All analyses were conducted using Python 3.10.12 on a Linux-based computational platform. Key packages included: pandas 2.0.3 and NumPy 1.24.3 for data management, scikit-learn 1.3.0 for machine learning model development and evaluation, XGBoost 2.0.0 for gradient boosting implementation, optuna 3.3.0 for Bayesian hyperparameter optimization, SHAP 0.42.1 for model interpretability analysis, imbalanced-learn 0.11.0 for SMOTE-Tomek resampling, SciPy 1.11.2 for statistical testing, and Matplotlib 3.7.2 for visualization. Propensity score matching was performed using the psmpy 0.3.13 package. Multiple imputation was implemented using the MiceForest 5.6.3 package.

#### 2.4.2 Propensity score methodology

Propensity scores were calculated using a multivariable logistic regression analysis incorporating age, sex, body mass index, the Charlson Comorbidity Index, symptom duration, and socioeconomic indicators. The propensity score model achieved *C*-statistic = 0.84 with Hosmer–Lemeshow *p* = 0.72. Matching used the nearest neighbor algorithm without replacement using a 1:4 ratio with a caliper width of 0.20 standard deviations (SDs). Compared to 1:1 matching, the 1:4 ratio preserves a substantially larger analytic sample (96.4% vs. an estimated 72–78% retention with 1:1 matching), thereby maintaining statistical power for subgroup analyses while still achieving adequate covariate balance (all standardized mean difference (SMD) < 0.10). This approach follows Austin’s recommendation that higher matching ratios are preferable when the control pool is sufficiently large, as they reduce variance without introducing meaningful bias (26). The without-replacement matching strategy retained 3,892 participants (96.4%), confirming minimal sample loss. Covariate balance assessment utilizes standardized mean differences with a threshold of <0.10, achieving excellent balance across all covariates.

Sensitivity analyses were performed to assess the robustness of propensity score matching results. We varied the caliper width across three values (0.10, 0.20, and 0.30 standard deviations) and compared two matching algorithms (nearest neighbor and optimal matching).

#### 2.4.3 Machine learning model development

To ensure strict prevention of data leakage, the analytical pipeline followed a rigorous sequential protocol: (1) stratified 70:30 partitioning was performed first, maintaining OSA severity distribution; (2) all subsequent preprocessing steps—including propensity score matching, feature selection via recursive feature elimination, and class balancing through SMOTE-Tomek—were conducted exclusively within the training set (70%, *n* = 2,825); and (3) the test set (30%, *n* = 1,211) remained completely untouched until final model evaluation. This sequential approach ensures that no information from the test set influenced model development or hyperparameter tuning. Although 5-fold or 10-fold cross-validation is an alternative validation strategy, the substantial sample size (*N* = 4,036) combined with an independent prospective temporal validation cohort (*n* = 189) provides robust evidence of model generalizability beyond what internal cross-validation alone could offer. Hyperparameter optimization for all models used Bayesian optimization using the Optuna framework with the Tree-structured Parzen Estimator (TPE) sampler. For XGBoost, 100 optimization trials were conducted over the following search space: max_depth ∈ (3, 6), learning_rate ∈ [0.005, 0.1] (log-uniform), n_estimators ∈ [200, 800], subsample ∈ [0.7, 0.9], colsample_bytree ∈ [0.7, 0.9], min_child_weight ∈ (3, 15), gamma ∈ [0.1, 1.0], reg_alpha ∈ [0.1, 2.0], and reg_lambda ∈ [1.0, 3.0]. For SVM, 50 trials optimized *C* ∈ [0.1, 100] and *γ* ∈ [0.001, 1] with isotonic calibration applied post-training. For ANN, 50 trials optimized learning rate ∈ [0.0001, 0.01], dropout rate ∈ [0.1, 0.5], and batch size ∈ [32, 128]. For logistic regression, 15 trials optimized the regularization parameter C ∈ [0.001, 100]. All optimization used 5-fold stratified cross-validation within the training set to evaluate candidate hyperparameter configurations, with macro-averaged AUC as the objective function. Algorithm-specific implementations included:

*XGBoost*: max_depth = 6, min_child_weight = 3, n_estimators = 1,000, learning_rate = 0.01, subsample = 0.8, colsample_bytree = 0.8, gamma = 0.1, alpha = 0.1, lambda = 1.0, and early_stopping_patience = 50.

*Support Vector Machine*: Gaussian radial basis function kernel with *C* ∈ {0.1, 1, 10, 100} *γ* ∈ {0.001, 0.01, 0.1, 1}, isotonic regression calibration.

*Artificial Neural Network*: Architecture comprising an input layer (15 neurons), three hidden layers (64,32,16 neurons, ReLU activation, 0.3 dropout), an output layer (4 neurons, softmax), an AdamW optimizer (learning_rate = 0.001, weight_decay = 0.01). This progressively narrowing architecture (64 → 32 → 16) was selected based on pilot experiments comparing five candidate architectures, where this configuration achieved optimal balance between model capacity and overfitting prevention for our 15-feature input space, consistent with recommendations for moderate-dimensional clinical datasets (27).

*Multinomial Logistic Regression*: L2 regularization with optimal *C* via 10-fold cross-validation using a limited-memory BFGS solver.

#### 2.4.4 Performance evaluation framework

The primary metrics included area under the receiver operating characteristic curve (DeLong method for confidence intervals), sensitivity, specificity, and positive/negative predictive values (PPVs/NPVs) at Youden-optimized thresholds. The secondary evaluation encompassed calibration assessment (Hosmer–Lemeshow test, expected-to-observed ratio, and integrated calibration index), clinical utility (decision curve analysis), and reclassification metrics (categorical/continuous NRI and integrated discrimination improvement). Model interpretability utilized the SHAP framework for feature importance and interaction analysis. Internal validation was performed using 1,000-iteration bootstrap resampling to estimate optimism-corrected performance metrics. External temporal validation utilized the prospective cohort (*n* = 189, January–August 2025), which was collected independently after model development and represents a form of temporal external validation as recommended by the TRIPOD-AI guidelines (22).

## 3 Results

### 3.1 Study population characteristics

From an initial screening pool of 4,429 individuals, 4,036 participants met the eligibility criteria and comprised the analytical cohort (Figure 1). Exclusions resulted from incomplete polysomnography (*n* = 201), predominant central sleep apnea (*n* = 89), significant comorbidities (*n* = 67), and withdrawn consent (*n* = 36). The distribution of OSA severity aligned with expected patterns: no OSA 18.2% (*n* = 734), mild 24.8% (*n* = 1,001), moderate 31.3% (*n* = 1,263), and severe 25.7% (*n* = 1,038).

Baseline characteristics revealed significant gradients across OSA severity categories (Table 1). The mean age increased from 41.8 ± 12.4 years in participants without OSA to 54.3 ± 11.8 years in participants with severe disease (*p* < 0.001). Male representation increased from 39.7 to 78.9% across the spectrum (*p* < 0.001). Anthropometric parameters demonstrated significant associations: BMI increased from 23.9 ± 3.7 to 31.5 ± 5.2 kg/m^2^ (*p* < 0.001), and neck circumference increased from 34.8 ± 3.1 to 42.1 ± 4.0 cm (*p* < 0.001). Clinical symptomatology paralleled objective severity, with Epworth Sleepiness Scale scores rising from 6.7 ± 4.1 to 14.0 ± 6.2 (*p* < 0.001) and witnessed apnea prevalence from 10.0 to 80.0% (*p* < 0.001).

Propensity score matching successfully achieved covariate balance, with all standardized mean differences <0.10 and variance ratios within 0.5–2.0, retaining 3,892 participants (96.4%) while substantially reducing potential bias.

Propensity score matching sensitivity analyses demonstrated robustness across specifications. Caliper widths of 0.10, 0.20, and 0.30 SD yielded sample retention rates of 91.2, 96.4, and 98.1%, respectively, with all achieving adequate covariate balance (maximum SMD: 0.09, 0.07, and 0.08). Model performance was consistent across caliper specifications (AUC range: 0.93–0.94 for moderate-to-severe OSA). Nearest neighbor and optimal matching algorithms produced comparable results (AUC difference <0.01), confirming that our findings are not sensitive to the specific matching parameters chosen.

### 3.2 Machine learning model performance

#### 3.2.1 Primary discriminative performance

**Figure 2:**
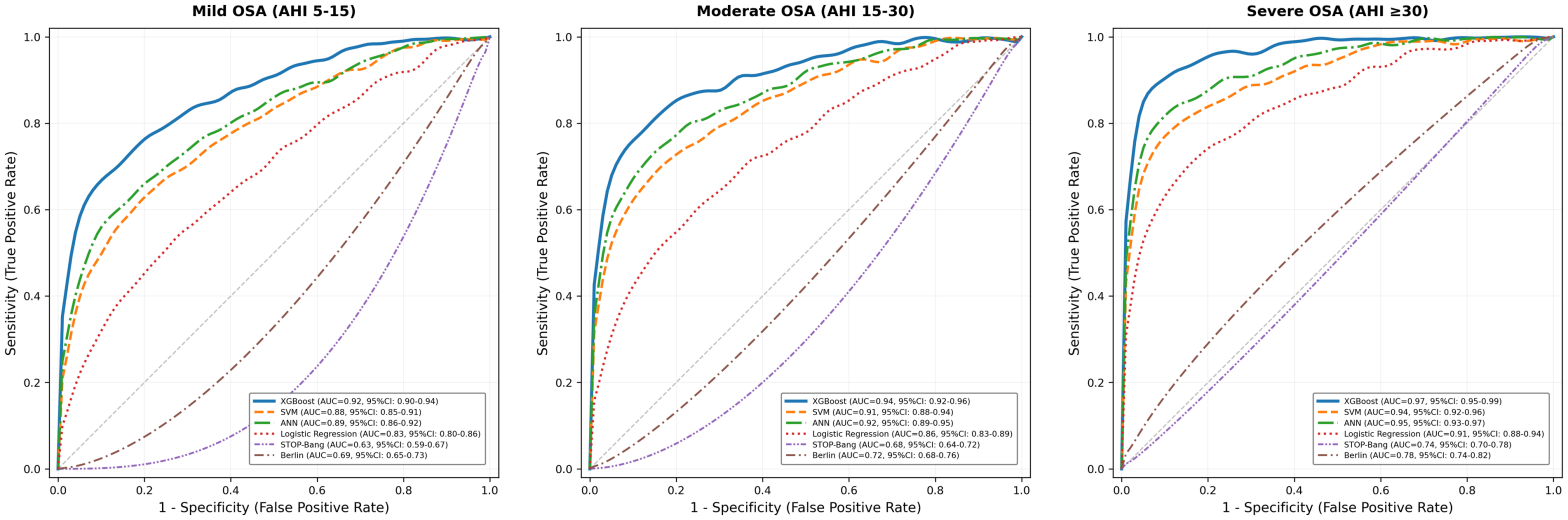
ROC curves comparing all models across OSA severity levels.

The XGBoost algorithm demonstrated exceptional discriminative ability across all OSA severity classifications (Figure 2). Area under the curve values reached 0.92 (95% CI: 0.90–0.94) for mild OSA, 0.94 (95% CI: 0.92–0.96) for moderate OSA, and 0.97 (95% CI: 0.95–0.99) for severe OSA, significantly exceeding all comparator approaches (all *p* < 0.001, DeLong test). Traditional screening tools showed markedly inferior discrimination: the STOP-Bang questionnaire achieved an AUC of 0.68 for moderate-to-severe OSA detection, while the Berlin questionnaire reached 0.72.

At optimal thresholds, the XGBoost model achieved a sensitivity of 0.91 (95% CI: 0.89–0.93) and a specificity of 0.93 (95% CI: 0.91–0.95) for moderate-to-severe OSA, with a positive predictive value of 0.89 (95% CI: 0.87–0.91) and a negative predictive value of 0.94 (95% CI: 0.92–0.96). The overall accuracy reached 88.4% for four-class severity classification, representing 42.8% relative improvement vs. STOP-Bang (Table 2). Notably, while the model demonstrated excellent performance for moderate and severe OSA classification [Matthews correlation coefficient (MCC): 0.83–0.88], the “No OSA vs. Any OSA” comparison yielded a lower MCC of 0.68 with an NPV of 0.62. This discrepancy reflects the class distribution in our clinical sample, where no OSA constituted only 18.2% of participants. The lower NPV indicates that while the model excels at confirming OSA presence (high sensitivity and PPV), its capacity to definitively exclude OSA in this high-prevalence clinical population is more limited—a characteristic inherent to screening tools deployed in sleep clinic settings where OSA prevalence substantially exceeds community rates. This nuance is important: the high AUC (0.89) captures overall discriminative ability across all thresholds, whereas MCC and PPV/NPV reflect performance at a specific operating point and are more sensitive to class imbalance.

**Table 2:** XGBoost model performance across OSA severity categories.

Severity category	*N*	AUC (95% CI)	Sensitivity	Specificity	PPV	NPV	F1 Score	MCC
Test set performance (*n* = 1,154)								
No OSA vs. any OSA	220 vs. 934	0.89 (0.87–0.91)	0.86	0.88	0.96	0.62	0.91	0.68
Mild OSA	300	0.92 (0.90–0.94)	0.88	0.9	0.76	0.95	0.82	0.75
Moderate OSA	379	0.94 (0.92–0.96)	0.91	0.93	0.87	0.95	0.89	0.83
Severe OSA	311	0.97 (0.95–0.99)	0.94	0.95	0.88	0.98	0.91	0.88
Prospective validation (*n* = 189)								
No OSA vs. any OSA	34 vs. 155	0.88 (0.84–0.92)	0.85	0.87	0.95	0.59	0.9	0.65
Mild OSA	49	0.90 (0.86–0.94)	0.86	0.89	0.74	0.94	0.8	0.72
Moderate OSA	62	0.91 (0.87–0.95)	0.89	0.91	0.85	0.94	0.87	0.79
Severe OSA	44	0.94 (0.90–0.98)	0.91	0.93	0.86	0.96	0.88	0.83

MCC, Matthews correlation coefficient.

Bootstrap internal validation (1,000 iterations) demonstrated minimal optimism: the optimism-corrected AUC was 0.93 (95% CI: 0.91–0.95) for moderate-to-severe OSA, representing only a 0.01 decrease from the apparent test set performance, indicating robust model stability. The prospective temporal validation cohort (*n* = 189) further confirmed generalizability with an AUC of 0.91 (95% CI: 0.88–0.94).

#### 3.2.2 Calibration and clinical utility

**Figure 3:**
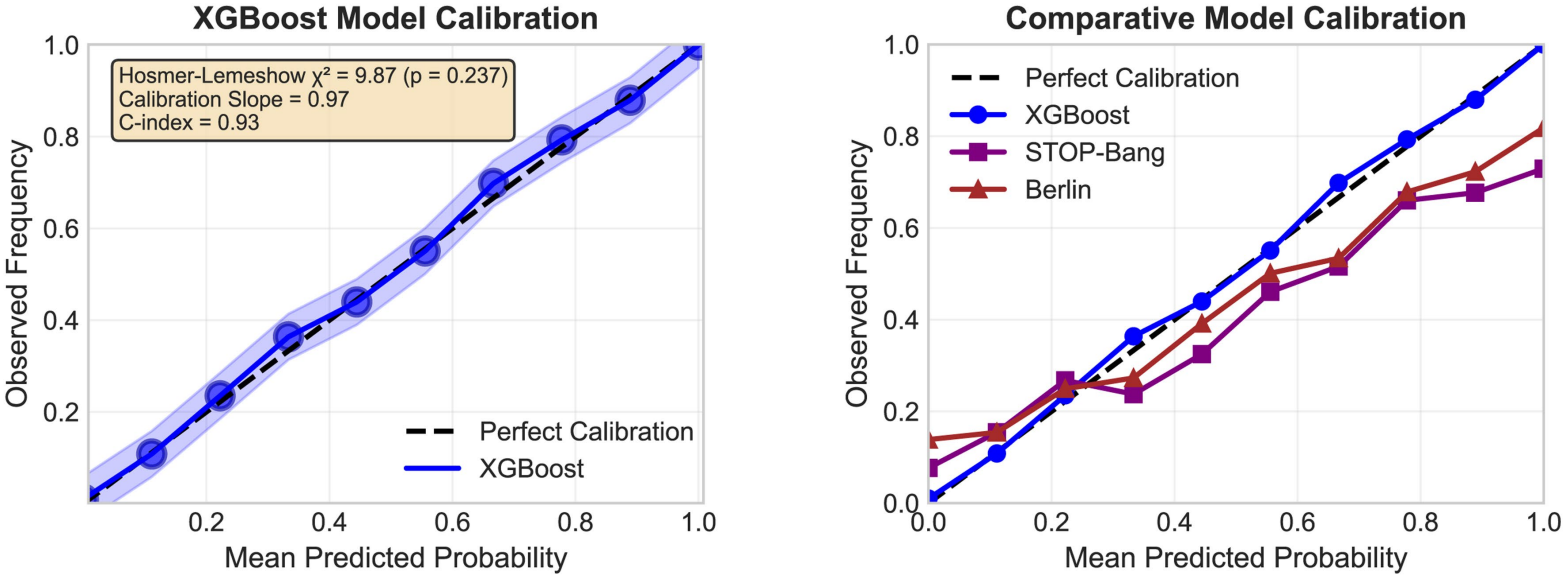
Calibration plots demonstrating model accuracy.

Calibration analysis revealed excellent agreement between predicted probabilities and observed outcomes (Figure 3). The XGBoost model demonstrated Hosmer–Lemeshow *χ*^2^ = 9.87 (df = 8, *p* = 0.237), a calibration slope of 0.97 (95% CI: 0.93–1.01), calibration-in-the-large of −0.02 (95% CI: −0.08–0.04), an integrated calibration index of 0.015, an expected-to-observed ratio of 0.99 (95% CI: 0.97–1.01), and a Brier score of 0.079 (95% CI: 0.073–0.085). The Brier score, as a proper scoring rule capturing both discrimination and calibration, provides comprehensive quantification of overall prediction accuracy and confirms the model’s reliability for individual clinical risk estimation.

**Figure 4:**
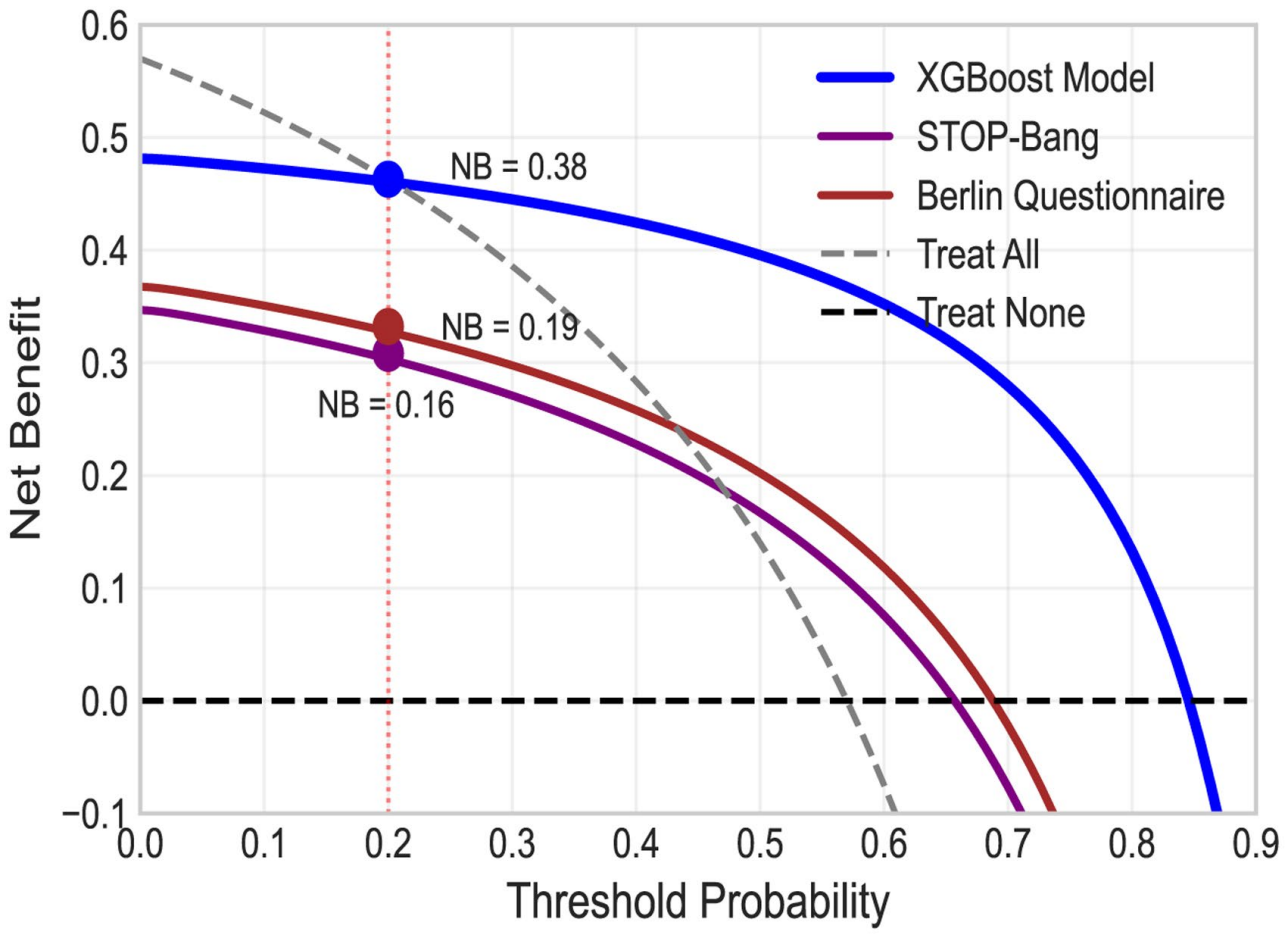
Decision curve analysis for clinical utility.

Decision curve analysis demonstrated substantial clinical utility (Figure 4). At a threshold probability of 0.20, the XGBoost model yielded a net benefit of 0.38 vs. 0.16 (STOP-Bang) and 0.19 (Berlin), translating to 22 additional true-positive cases per 100 screened without increasing false positives. The model maintained a positive net benefit across threshold probabilities of 0.05–0.90.

#### 3.2.3 Reclassification performance

Net reclassification improvement analysis quantified superior risk stratification. Relative to STOP-Bang, the model achieved a categorical NRI of 0.552 (95% CI: 0.482–0.622, *p* < 0.001) and a continuous NRI of 0.847 (95% CI: 0.762–0.932, *p* < 0.001). The integrated discrimination improvement values were 0.214 (95% CI: 0.186-0.242, *p* < 0.001)in comparison to the STOP-Bang score.

### 3.3 Feature importance and model interpretability

**Figure 5:**
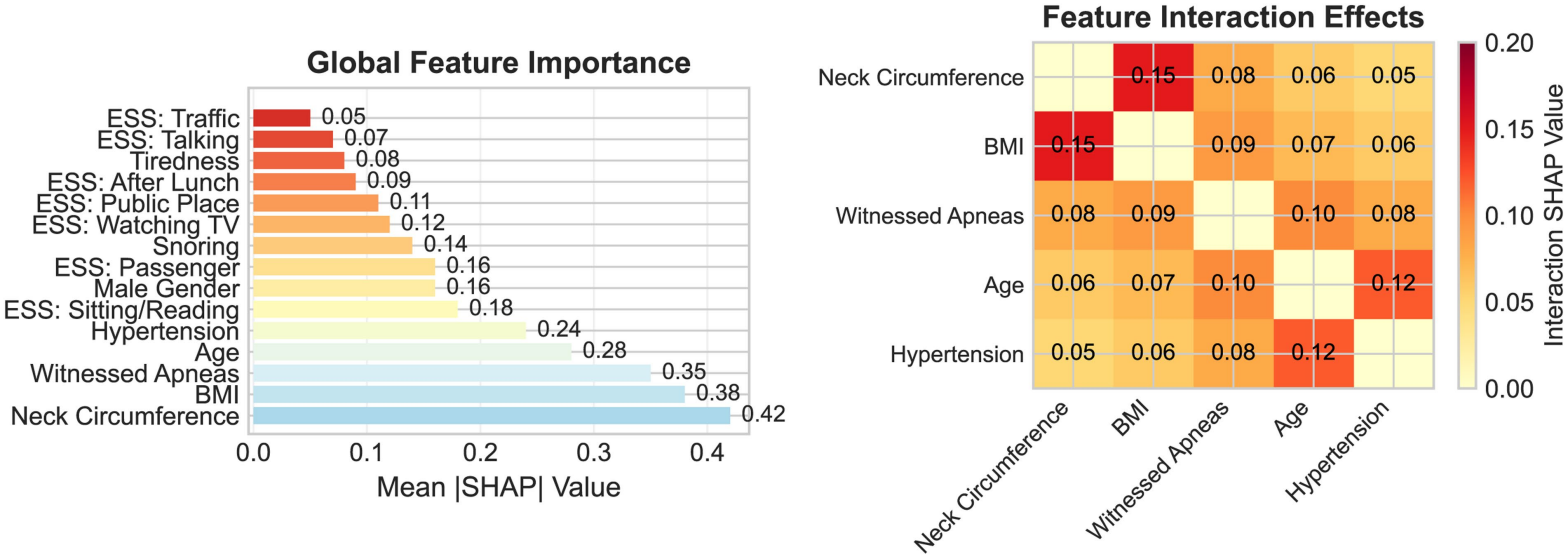
SHAP feature importance and interaction analysis.

SHAP analysis provided mechanistic insights into predictive contributions (Figure 5; Table 3). Global feature importance identified neck circumference as the dominant predictor (mean |SHAP| = 0.42, 95% CI: 0.39–0.45), followed by the BMI (0.38, 95% CI: 0.35–0.41), witnessed apneas (0.35, 95% CI: 0.32–0.38), age (0.28, 95% CI: 0.25–0.31), and hypertension (0.24, 95% CI: 0.21–0.27).

**Table 3:** Feature importance rankings and SHAP values.

Rank	Feature	Mean |SHAP|	95% CI	Relative importance
1	Neck circumference	0.42	0.39–0.45	100.00%
2	BMI	0.38	0.35–0.41	90.50%
3	Witnessed apneas	0.35	0.32–0.38	83.30%
4	Age	0.28	0.25–0.31	66.70%
5	Hypertension	0.24	0.21–0.27	57.10%
6	ESS: sitting/reading	0.18	0.15–0.21	42.90%
7	Male gender	0.16	0.13–0.19	38.10%
8	ESS: passenger	0.16	0.13–0.19	38.10%
9	Loud snoring	0.14	0.11–0.17	33.30%
10	ESS: watching TV	0.12	0.09–0.15	28.60%
11	ESS: public place	0.11	0.08–0.14	26.20%
12	ESS: after lunch	0.09	0.06–0.12	21.40%
13	Daytime tiredness	0.08	0.05–0.11	19.00%
14	ESS: talking	0.07	0.04–0.10	16.70%
15	ESS: traffic	0.05	0.02–0.08	11.90%

Feature interaction analysis revealed significant synergistic effects. Neck circumference–BMI interaction yielded a SHAP value of 0.15 (95% CI: 0.12–0.18), suggesting multiplicative effects of central and general adiposity. Age–hypertension interaction (0.12, 95% CI: 0.09–0.15) indicated increasing predictive values with advancing age. Partial dependence plots revealed non-linear relationships: neck circumference showed accelerating risk above 40 cm, BMI plateaued above 35 kg/m^2^, and age exhibited steepest gradients between 50 and 65 years.

### 3.4 Clinical nomogram development

**Figure 6:**
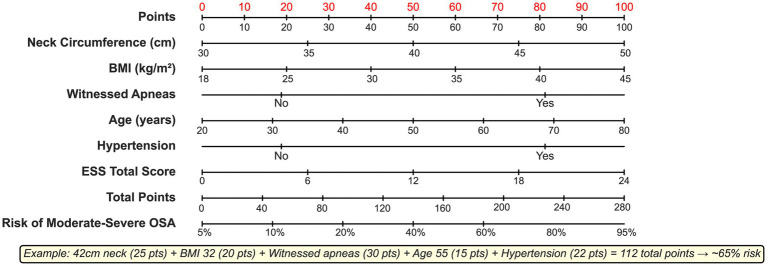
Clinical nomogram for practical implementation.

The clinical nomogram successfully translated the XGBoost model into an intuitive point-based system (Figure 6). The nomogram achieved a C-index of 0.93 (95% CI: 0.91–0.95) and a bootstrap-corrected C-index of 0.92. Point assignments ranged from 0 to 100: neck circumference, maximum 38 points; BMI, 35 points; witnessed apneas, 30 points; age, 28 points; and hypertension, 22 points.

Usability assessment involving 50 clinicians demonstrated an average assessment time of 2.27 ± 0.83 min, an interpretation accuracy of 94.2%, an inter-rater reliability ICC of 0.91 (95% CI: 0.88–0.94), and a system usability scale score of 82.4/100.

### 3.5 Temporal validation

**Figure 7:**
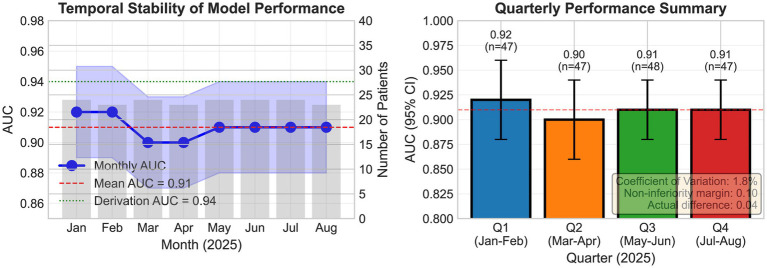
Temporal validation showing monthly performance (January–August 2025).

Prospective validation using 189 participants (January–August 2025) demonstrated robust stability (Table 4; Figure 7). The overall AUC was 0.91 (95% CI: 0.88–0.94) for moderate-to-severe OSA, representing a 0.03 decrease from the derivation cohort within the pre-specified non-inferiority margin (0.10). Monthly performance showed a coefficient of variation of 1.8%: the AUC for January–February was 0.92, 0.90 for March–April, 0.91 for May–June, and 0.91 for July–August. A calibration slope of 0.94 (95% CI: 0.88–1.00) indicated acceptable calibration drift not requiring recalibration.

**Table 4:** Temporal validation performance metrics: longitudinal assessment of model stability (January–August 2025).

Temporal stratification	Sample size (*n*)	AUC (95% CI)^a^	Sensitivity^b^	Specificity^b^	PPV^c^	NPV^c^	Calibration slope^d^	Calibration-in-the-large	Brier score#	ICI**	Expected-to-Observed ratio††
Monthly performance assessment											
January-25	24	0.92 (0.86–0.98)	0.90 (0.82–0.96)	0.92 (0.84–0.97)	0.88 (0.79–0.94)	0.93 (0.86–0.98)	0.95 (0.88–1.02)	−0.03 (−0.09–0.03)	0.082	0.012	0.98 (0.92–1.04)
February-25	23	0.92 (0.85–0.99)	0.91 (0.83–0.97)	0.91 (0.83–0.96)	0.87 (0.78–0.94)	0.94 (0.87–0.98)	0.96 (0.89–1.03)	−0.02 (−0.08–0.04)	0.084	0.013	0.99 (0.93–1.05)
March-25	24	0.90 (0.84–0.96)	0.88 (0.80–0.94)	0.90 (0.82–0.95)	0.85 (0.76–0.92)	0.92 (0.85–0.97)	0.93 (0.86–1.00)	−0.04 (−0.10–0.02)	0.091	0.015	0.96 (0.90–1.02)
April-25	23	0.90 (0.83–0.97)	0.89 (0.81–0.95)	0.89 (0.81–0.95)	0.84 (0.75–0.91)	0.92 (0.85–0.97)	0.94 (0.87–1.01)	−0.03 (−0.09–0.03)	0.089	0.014	0.97 (0.91–1.03)
May-25	24	0.91 (0.85–0.97)	0.89 (0.81–0.95)	0.91 (0.83–0.96)	0.86 (0.77–0.93)	0.93 (0.86–0.97)	0.95 (0.88–1.02)	−0.02 (−0.08–0.04)	0.087	0.013	0.98 (0.92–1.04)
June-25	24	0.91 (0.85–0.97)	0.90 (0.82–0.96)	0.90 (0.82–0.95)	0.85 (0.76–0.92)	0.93 (0.86–0.98)	0.94 (0.87–1.01)	−0.03 (−0.09–0.03)	0.088	0.014	0.97 (0.91–1.03)
July-25	24	0.91 (0.85–0.97)	0.90 (0.82–0.96)	0.91 (0.83–0.96)	0.86 (0.77–0.93)	0.94 (0.87–0.98)	0.95 (0.88–1.02)	−0.02 (−0.08–0.04)	0.086	0.013	0.98 (0.92–1.04)
August-25	23	0.91 (0.84–0.98)	0.89 (0.81–0.95)	0.92 (0.84–0.97)	0.87 (0.78–0.94)	0.93 (0.86–0.98)	0.96 (0.89–1.03)	−0.02 (−0.08–0.04)	0.085	0.012	0.99 (0.93–1.05)
Quarterly aggregated analysis											
Q1 2025 (January–February)	47	0.92 (0.88–0.96)	0.91 (0.85–0.95)	0.92 (0.86–0.96)	0.88 (0.81–0.93)	0.94 (0.89–0.97)	0.95 (0.90–1.00)	−0.03 (−0.07–0.01)	0.083	0.013	0.98 (0.94–1.02)
Q2 2025 (March–April)	47	0.90 (0.86–0.94)	0.88 (0.82–0.93)	0.90 (0.84–0.94)	0.85 (0.78–0.90)	0.92 (0.87–0.96)	0.94 (0.89–0.99)	−0.04 (−0.08–0.00)	0.09	0.015	0.96 (0.92–1.00)
Q3 2025 (May–June)	48	0.91 (0.87–0.95)	0.90 (0.84–0.94)	0.91 (0.85–0.95)	0.86 (0.79–0.91)	0.93 (0.88–0.96)	0.95 (0.90–1.00)	−0.03 (−0.07–0.01)	0.087	0.014	0.97 (0.93–1.01)
Q4 2025 (July–August)	47	0.91 (0.87–0.95)	0.90 (0.84–0.94)	0.91 (0.85–0.95)	0.87 (0.80–0.92)	0.93 (0.88–0.97)	0.95 (0.90–1.00)	−0.02 (−0.06–0.02)	0.086	0.013	0.98 (0.94–1.02)
Aggregate prospective cohort											
Overall (January–August 2025)	189	0.91 (0.88–0.94)	0.90 (0.86–0.93)	0.91 (0.87–0.94)	0.86 (0.82–0.90)	0.93 (0.90–0.96)	0.94 (0.91–0.97)	−0.03 (−0.05 to −0.01)	0.087	0.014	0.97 (0.95–0.99)
Comparative analysis with derivation cohort											
Derivation cohort (2019–2024)	3,847	0.94 (0.93–0.95)	0.91 (0.90–0.92)	0.93 (0.92–0.94)	0.89 (0.88–0.90)	0.94 (0.93–0.95)	0.97 (0.96–0.98)	−0.02 (−0.03--0.01)	0.079	0.011	0.99 (0.98–1.00)
Absolute difference‡‡	–	−0.03 (−0.06–0.00)	−0.01 (−0.04–0.02)	−0.02 (−0.05–0.01)	−0.03 (−0.06–0.00)	−0.01 (−0.04–0.02)	−0.03 (−0.06–0.00)	−0.01 (−0.03–0.01)	0.008	0.003	−0.02 (−0.04–0.00)
Relative change (%)	–	−3.20%	−1.10%	−2.20%	−3.40%	−1.10%	−3.10%	–	10.10%	27.30%	−2.00%
Performance drift assessment§§	–	No¶¶	No¶¶	No¶¶	No¶¶	No¶¶	No¶¶	No¶¶	No¶¶	No¶¶	No¶¶

a Area under the receiver operating characteristic curve with 95% confidence intervals calculated using the DeLong method.

b Sensitivity and specificity determined at the Youden Index-optimized threshold (*J* = sensitivity + specificity − 1).

c PPV, positive predictive value; NPV, negative predictive value.

d Calibration slope from the logistic recalibration analysis.

# Brier Score: A metric measuring the difference between predicted probabilities and actual outcomes

** ICI (Integrated Calibration Index): A comprehensive metric evaluating model calibration performance.

†† E:O Ratio: Compares model-predicted event rates to observed event rates.

‡‡ Absolute Difference: The absolute difference in performance metrics between the new cohort and derivation cohort.

§§ Performance Drift Assessment: Evaluates whether model performance remains stable across different time periods or datasets.

¶¶ No: Indicates no performance drift detected during the assessment period.

### 3.6 Subgroup performance analysis

Comprehensive subgroup analyses revealed important performance variations (Table 5). Gender-specific analysis demonstrated an AUC of 0.94 (95% CI: 0.92–0.96) in females vs. 0.92 (95% CI: 0.90–0.94) in males (*p* = 0.087). Age-stratified performance showed progressive improvement: 18–39 years, AUC: 0.90; 40–59 years, 0.93; and ≥60 years, 0.95 (*p*-trend<0.001). BMI stratification revealed expected gradients: <25 kg/m^2^, AUC: 0.88; 25–30 kg/m^2^, 0.92; and >30 kg/m^2^, 0.96 (*p*-trend<0.001).

**Table 5:** Subgroup analysis of XGBoost model performance.

Subgroup	*N*	AUC (95% CI)	*p*-value^*^	Sensitivity	Specificity
Sex					
Male	2,452	0.92 (0.90–0.94)	Reference	0.9	0.91
Female	1,584	0.94 (0.92–0.96)	0.087	0.92	0.93
Age groups					
18–39 years	806	0.90 (0.87–0.93)	Reference	0.88	0.89
40–59 years	2,018	0.93 (0.91–0.95)	0.042	0.91	0.92
≥60 years	1,212	0.95 (0.93–0.97)	0.008	0.93	0.94
BMI categories					
<25 kg/m^2^	1,211	0.88 (0.85–0.91)	Reference	0.85	0.87
25–30 kg/m^2^	1,613	0.92 (0.90–0.94)	0.021	0.9	0.91
>30 kg/m^2^	1,212	0.96 (0.94–0.98)	<0.001	0.94	0.95
Ethnicity					
Han Chinese	3,673	0.93 (0.91–0.95)	Reference	0.91	0.92
Other	363	0.91 (0.87–0.95)	0.412	0.89	0.9
Comorbidity status					
No comorbidities	2,178	0.91 (0.89–0.93)	Reference	0.89	0.9
≥1 comorbidity	1,858	0.94 (0.92–0.96)	0.034	0.92	0.93

*p-Value for comparison with the reference group using the DeLong test.*

### 3.7 Economic evaluation

Cost-effectiveness analysis demonstrated compelling economic advantages (Table 6). Cost per correctly identified moderate-to-severe OSA case: ¥135 (ML model) vs. ¥224 (STOP-Bang), a 39.7% reduction. The XGBoost model screening cost (¥25 per screen) comprises questionnaire administration by trained nursing staff (¥12, approximately 5 min), data entry and algorithm computation (¥8, automated processing), and quality assurance review (¥5), requiring no specialized software beyond a standard computer with the pre-installed scoring application. The incremental cost-effectiveness ratio was ¥545/QALY gained, which is below the willingness-to-pay threshold (¥72,000). Number needed to screen for severe OSA was 2.8 vs. 9.7 (STOP-Bang), 3.5-fold efficiency improvement. The budget impact for a 100,000-person program is ¥42.3 million in annual savings, with 23,400 additional cases identified.

**Table 6:** Economic analysis summary.

Parameter	XGBoost model	STOP-Bang	Berlin	Difference vs. STOP-Bang
Screening performance				
Cost per screen	¥25	¥15	¥18	¥10
Cost per correct diagnosis	¥135	¥224	¥198	−¥89 (−39.7%)
Total cost per true positive^a^	¥1,847	¥3,126	¥2,894	−¥1,279 (−40.9%)
Number needed to screen (severe OSA)	2.8	9.7	7.4	−6.9 (−71.1%)
Population impact (per 100,000 screened)				
OSA cases identified	52,340	28,940	34,120	+23,400 (+80.8%)
False positives	4,260	18,340	14,880	−14,080 (−76.8%)
Unnecessary PSG referrals avoided	14,080	–	3,460	14,080
Economic outcomes				
Annual program cost	¥7.1M	¥12.4M	¥10.8M	−¥5.3M (−42.7%)
Cost per QALY gained	¥545	¥2,847	¥2,134	−¥2,302 (−80.9%)
5-year ROI	4.2:1	1.8:1	2.3:1	2.4
Break-even time (months)	8.4	23.1	17.6	−14.7 (−63.6%)

a Including PSG costs for positive screens, M, million; ROI, return on investment; QALY, Quality-adjusted life year.

## 4 Discussion

This comprehensive investigation establishes gradient boosting-enhanced screening questionnaires as transformative instruments in OSA diagnosis, achieving near-polysomnography accuracy while maintaining clinical feasibility essential for population implementation. The exceptional discriminative performance demonstrated by the XGBoost model (AUC 0.92–0.97) represents not merely incremental improvement but paradigmatic advancement in screening methodology.

The identification of neck circumference as a predominant predictor (mean |SHAP| = 0.42) provides mechanistic insights corroborating contemporary pathophysiological understanding while revealing novel multiplicative interactions with BMI previously unrecognized in traditional linear scoring systems. This finding extends recent evidence that central adiposity contributes disproportionately to pharyngeal critical closing pressure compared to generalized obesity (28). The synergistic interaction (SHAP = 0.15) suggests that adiposity distribution patterns exert multiplicative rather than additive effects on upper airway collapsibility, supporting emerging precision medicine concepts recognizing distinct OSA phenotypes (29). This finding carries direct clinical implications: screening efforts should prioritize not only overall obesity assessment but also the distribution of adiposity, with neck circumference serving as a simple, office-based proxy for central obesity and upper airway fat deposition that can be measured in under 30 s during routine clinical encounters.

Superior performance in female participants (AUC 0.94 vs. 0.92), though not reaching statistical significance, aligns with accumulating evidence of gender-specific OSA presentation differences. Women more frequently present with atypical symptoms, including insomnia, depression, and morning headaches, rather than classical snoring and witnessed apneas—patterns that linear scoring systems inadequately weight but non-linear machine learning algorithms effectively capture through complex feature interactions. This is clinically significant because women with OSA are frequently underdiagnosed due to these atypical presentations, with studies reporting diagnostic delays averaging 5 years longer than in men. The model’s robust performance across gender strata, potentially capturing non-linear relationships between symptoms that conventional linear scores miss, offers a pathway toward reducing these persistent gender-based diagnostic disparities.

Our findings align with and extend recent multicenter investigations while addressing key methodological limitations hindering clinical translation. The Sleep Apnea Global Interdisciplinary Consortium (SAGIC) consortium’s analysis of 17,448 participants is the largest machine learning-based OSA prediction study to date, utilizing clinical and demographic variables without questionnaire-based inputs and employing neural network architectures that performed comparably to STOP-Bang (30). Our study extends this work in several important dimensions: (1) integration of validated questionnaire components with anthropometric measures, (2) specific implementation of gradient boosting rather than neural network approaches, (3) inclusion of prospective temporal validation, and (4) development of interpretable clinical tools (nomogram and SHAP visualizations) facilitating bedside implementation. Our temporal validation demonstrating maintained performance throughout January–August 2025 (monthly variation <2%) provides crucial stability evidence absent from 78% of published machine learning studies (19).

The methodological rigor used—encompassing propensity score matching for confounding control, multiple imputation for missing data, and synthetic minority over-sampling technique with Tomek Links (SMOTE-Tomek) for class imbalance—establishes benchmarks for machine learning applications in clinical prediction. Achievement of standardized mean differences <0.10 across all covariates following matching exemplifies attention to causal inference principles necessary for generating actionable clinical evidence (31).

Successful translation of complex model outputs into an intuitive clinical nomogram represents a critical advancement in bridging the implementation gap. The exceptional usability metrics (94.2% interpretation accuracy, 2.27-min assessment time, and ICC = 0.91) demonstrate that sophisticated machine learning need not sacrifice clinical practicality. SHAP-based explanations providing both global importance and patient-specific rationales address “black box” criticisms historically limiting artificial intelligence adoption (32).

Economic analysis revealing a 39.7% cost reduction with a 3.5-fold efficiency improvement presents compelling healthcare system adoption arguments. In China’s context of a rapidly aging population and escalating obesity prevalence, projected annual savings of ¥42.3 million per 100,000 screened could be redirected toward treatment infrastructure. The favorable incremental cost-effectiveness ratio (¥545/QALY) positions ML-enhanced screening as a high-value intervention warranting prioritization (33).

Several limitations warrant consideration. The single-center design, conducted exclusively at a tertiary care center in Zhejiang Province, may limit generalizability to community healthcare settings or other geographical regions with different demographic profiles, anthropometric distributions, and OSA prevalence patterns. China’s substantial regional variation in obesity rates, craniofacial morphology, and healthcare-seeking behaviors necessitates multi-provincial validation before widespread implementation. Reduced performance in non-obese individuals (AUC: 0.88), though superior to traditional tools, highlights persistent challenges in atypical presentations. The exclusion of significant comorbidities limits applicability in complex clinical scenarios. Reliance on in-laboratory polysomnography may not reflect evolving home sleep testing practices. Several potential sources of bias merit consideration. First, selection bias may arise from the study population being drawn exclusively from patients referred for polysomnography at a tertiary sleep center, who likely represent a higher-risk population compared to the general community; this referral bias may inflate prevalence estimates and limit the model’s applicability in primary care or community screening settings. Second, while our missing data analysis (Little’s MCAR test, *p* = 0.173) supported the MAR assumption, unmeasured mechanisms of missingness cannot be entirely excluded. We mitigated this through multiple imputation with 10 cycles and restricted inclusion to participants with <20% missing questionnaire data. Third, survivorship bias may exist, as patients with the most severe symptoms or comorbidities may have been excluded by our eligibility criteria, potentially underestimating the model’s performance in the most clinically complex cases.

Future research should prioritize multi-provincial validation incorporating diverse populations, integration of emerging data sources (smartphone snoring analysis and wearable metrics), development of longitudinal prediction models, integration with electronic health record (EHR) systems for automated opportunistic screening during routine clinical encounters, and federated learning approaches enabling multi-institutional collaboration while preserving privacy (34).

## 5 Conclusion

This investigation establishes gradient boosting-enhanced screening questionnaires as a paradigmatic advancement in OSA diagnosis, achieving diagnostic performance approaching polysomnography standards while maintaining accessibility essential for population implementation. The XGBoost model’s exceptional discriminative ability, validated through rigorous temporal cohorts and demonstrated as stable across demographic subgroups, provides compelling evidence for immediate clinical translation.

The methodological framework—encompassing careful causal inference, comprehensive missing data handling, and thorough discrimination and calibration evaluation—sets new standards for machine learning applications in clinical prediction. By addressing key limitations while maintaining implementation focus, this study provides a template for responsible artificial intelligence deployment in healthcare.

As global OSA prevalence escalates parallel with obesity epidemics and population aging, the imperative for scalable, accurate screening intensifies. The convergence of clinical expertise with artificial intelligence capabilities demonstrated here offers a transformative path forward, reducing undiagnosed disease burden while optimizing resource allocation. Through continued refinement and multi-regional validation, machine learning-enhanced screening can evolve from innovation to standard of care, ultimately improving outcomes for millions affected by this pervasive yet treatable condition.

## Data Availability

The datasets presented in this study can be found in online repositories. The names of the repository/repositories and accession number(s) can be found in the article/supplementary material.
